# Relationship between income inequality, socioeconomic development, vulnerability index, and maternal mortality in Brazil, 2017

**DOI:** 10.1186/s12889-021-11861-y

**Published:** 2021-10-12

**Authors:** Maria do Socorro Candeira Costa, Francisco Winter dos Santos Figueiredo

**Affiliations:** Epidemiology and Data Analysis Laboratory, FMABC University Center, Santo André, Brazil

**Keywords:** Maternal mortality, Socioeconomic factors, Basic health indicators

## Abstract

**Background:**

Public health recognizes that health conditions depend on factors related to the development patterns income distribution, degree of poverty, working conditions, among other social determinants. The objective of this study was to analyze the association of maternal mortality with the Human Development Index (HDI), Gini Index, Income per capita, and the Social Vulnerability.

**Method:**

The study analyzed the relationship between MMR and socioeconomic indicators in the 26 federative units and the Federal District of Brazil, in 2017. The socioeconomic indicators used in the study were: HDI, Gini Index, Income per capita, and SVI. Crude and adjusted linear regression were performed between maternal mortality and socioeconomic indicators.

**Results:**

When analyzing which socioeconomic determinants that are related to maternal mortality ratio rates, a higher per capita income positive effect was observed for lower MMR (β = − 150.8; CI 95% -289.9 to − 11.7; *r*^2^ = 0.17; *p* = 0.035), as well as a trend of higher MMR in relation to the SVI (β = 97.7; CI 95% -12.2 to 207.6; *r*^2^ = 0.12; *p* = 0.079). In model found by the stepwise forward selections, only the per capita income was um index related to less RMM (β = − 0.02; CI 95% -0.05 to − 0.002; *r*^2^ = 0.15; *p* = 0.028).

**Conclusion:**

The findings showed that the per capita income has a negative association MMR in the different states of Brazil, but seems canceled because of the other socioeconomic determinants related to the poor live conditions.

## Background

According to the World Health Organization (WHO), most people’s health problems are attributable to the social and economic conditions in which they live [1]. There is a vast amount of literature showing the association between income inequality and health situation, with very strong evidence of better health conditions in societies with more balanced income distribution [2, 3].

Public health recognizes that health conditions depend on factors related to the national development patterns income distribution, degree of poverty, working conditions, food and nutrition, sanitation, leisure, economic growth pattern, among other social determinants. For this reason, health has been increasingly incorporated into the development program in Brazil [4].

Among the most important health indicators of the world population are maternal and neonatal mortality rates. In Brazil, there has been an important decline in these rates in the last two decades, however, they remain with very high and still unacceptable values. The main causes of maternal and neonatal mortality include low birth weight, prematurity, hypertensive diseases, sepsis, complications of childbirth, puerperium and abortion [5].

The Maternal Mortality Ratio (MMR) index is an indicator that reflects the quality of health care of women in reproductive age. High MMR values are associated with the unsatisfactory provision of health services to this segment of the population, from family planning and prenatal care, to childbirth and postpartum care. Unsatisfactory health care from prenatal to childbirth and postpartum. The MMR is not only an indicator of health, but also an indicator of socioeconomic inequalities, because it is higher in underdeveloped or developing areas when compared to developed areas [6, 7].

In Brazil, maternal mortality is a serious public health problem due to its high incidence. Data from the Ministry of Health indicate that the MMR in 2016 was 64.4 deaths per 100,000 live births [7, 8]. These figures are considered high, as developed countries reach 12 deaths per 100,000, and the World Health Organization considers an MMR of up to 20 deaths per 100,000 live births acceptable [1, 6].

The study of social indicators is greatly important to interpret social reality, contributing to the analysis, formulation and implementation of public policies [9] Some of the important socioeconomic indicators used for this purpose are: Human Development Index (HDI), Income per capita, GINI Index and Social Vulnerability Index (SVI). These indicators are measures which through socioeconomic variables are used to understand the social reality.

The HDI expresses a measure of progress in three basic dimensions of human development: income, education and health. These dimensions measure a society’s opportunities to live long and healthy life and to have access to knowledge and resources that guarantee it a decent standard of living. The HDI has captured human progress in only one number and has served as a tool of excellence that guides public debates on national priorities [10, 11].

The Gini Index is used to measure how a country’s (or other administrative or geographical level) income is distributed among its population, showing the degree of income concentration in a given group. He points out the difference between the incomes of the poorest and the richest. The Gini coefficient was identified as a superior tool for measuring the inequality of a society [12, 13].

The per capita income also is an important method for measures the life conditions in a place, it assign the ability of the inhabitants of a given place to ensure a standard of living capable of ensuring their basic needs, such as water, food and housing [14].

Additionally, the SVI is an indicator that aims to measure the access or absence or insufficiency of this access to resources that should be available to every citizen, and that their possession or deprivation determines the conditions of well-being of populations in contemporary societies. The social vulnerability expressed in the SVI results from the selection of sixteen indicators that are organized in three dimensions: I) urban infrastructure; (II) human capital; and III) income and work. The higher the SVI of a territory, the greater its social vulnerability and, therefore, the greater the precariousness of the living conditions of its population. The SVI has been used in many studies as a support for the identification of people who are in cases of vulnerability [15, 16].

The HDI, Income per capita, GINI Index and SVI, aggregates 21 dimensions in total, summarizing complex and multidimensional issues of social reality. Differences in socioeconomic conditions can affect the population’s health conditions, and knowing that Brazil is a country with historical and expressive regional inequalities, characterized by enormous disparity between basic levels of economic, and social development among the 27 states of the federation [17], we questioned how these different socioeconomic indicators are associated with maternal mortality.

Many other indicators are important to analyze the association with maternal mortality, including health behavior and biological factors, but in this study the focus is on socioeconomic indicators. WHO recognizing socioeconomic and demographic characteristics as the most structural determinants of health and mortality [18]. We also highlight the focus on socioeconomic indicators considering the results of some studies that indicate that socioeconomic inequalities may have a more important role in women’s health compared to men’s health, as they shape access to services and resources that are especially central in women’s lives [19, 20].

The HDI, Gini Index, Income per capita and SVI indicators were chosen because they are able to present information of different dimensions of the socioeconomic conditions of Brazilian states, considering that they measure these conditions in their most varied aspects, that is: income inequality, ability to acquire goods and services and health conditions, education and urban infrastructure.

Considering the hypothesis that maternal mortality is not homogeneously distributed in Brazil and that maternal death is related to the socioeconomic level of the population, the aim of this study was to analyze the association between maternal mortality and HDI, Gini Index, Income per capita, and SVI.

## Methods

### Study design

This is an ecological study performed in 2020 with 2017 data.

### Geographical and temporal delimitation

The data used refer to the 27 federal units (26 federal units and the Federal District) in Brazil and were collected in 2017.

### Data source

The information on maternal death was extracted from the Health System Performance Evaluation Project (PROADESS) portal, available at the electronic address (https://www.proadess.icict.fiocruz.br), which is based on the Mortality Information System and has a record of data collected from the standardized death certificate and the Living Birth Information System. This system gathers epidemiological information on informed births throughout the country and has a record of data collected from the standardized declaration of live birth.

HDI, Gini Index, Income per capita, and SVI data were collected from the Institute of Applied Economic Research (IPEA) in 2017, available through the Social Vulnerability Atlas, at the electronic address (http://ivs.ipea.gov.br/index.php/pt/). IPEA is a federal public foundation linked to the Ministry of Economy that aims to conduct social and economic research and has a demographic, economic and geographic database available for Brazilian regions, states, and cities [15].

Access to the PROADESS and IPEA databases for data collection for this research took place in March 2020. The RMM and HDI, per capita income, GINI and IVS data are made available in open format in these databases.

### Exposure variables

Four socioeconomic indicators were considered as exposure variables: Gini Index, HDI, Income per capita, and SVI.

The Gini index assesses the level of income inequality between the residents of a locality and varies from 0 (no income inequality) to 1 (total income inequality) [13, 21].

The HDI used in this study is the result of the adaptation of the HDI to sub-national levels that has been used in several countries. Brazil adapted the global HDI methodology to calculate the Municipal HDI (MHDI) of the 5565 Brazilian cities, using the same process to obtain the HDI of the states. Minimum and maximum values are used to calculate the indicators of the three global HDI dimensions (income, education and health). The performance of each indicator varies from zero (minimum value) to 1 (maximum value), classifying the countries into four groups: countries with low human development (HDI less than 0.550), with medium human development (HDI between 0.550 and 0.699), with high human development (HDI between 0.700 and 0.799), and with very high human development (above 0.800) [10].

The per capita income indicator is calculated by adding the income of all residents and dividing it by the number of residents, including people without income records [10].

The SVI highlights different situations indicative of exclusion and social vulnerability in the Brazilian territory. The SVI is an index that varies between 0 and 1. The closer to 1, the greater the social vulnerability [15].

### Outcome variable

The MMR of the 27 states of the federation, collected from PROADESS as a quantitative variable was measured as an outcome and represents the risk of deaths from causes related to pregnancy, childbirth or puerperium, being an indicator of the quality of care in a community. MMR is calculated as the ratio of the number of women’s deaths caused by pregnancy, childbirth and puerperium (in the numerator) and the number of live births (in the denominator) [22].

### Data analysis

The MMR and socioeconomic indicators were described for each federative unit. Crude and adjusted linear regression were performed between maternal mortality and socioeconomic indicators. Two models were estimated to assess the relationship of the exposure variables and MMR to understand what model is better for understanding according to our objectives. First, we included all variables in model, estimating the adjusted R-square, and respective *p*-value and slope with 95% confidence interval. Finally, we used the follow methods for variable selection to found the variables related with the MMR: the stepwise forward, stepwise backward and both methods together. The criteria for adjustment of the regressions were the association between the variables, measured by the *p*-value of linear regression less the 0.20, and the command “sw” was used. For all analysis were describing the slope and the respective 95% confidence interval (CI 95%), predictive capacity (adjusted *r*^2^), and p-value. The significance level was 5%. The Stata® 11.0 software was used for the analyses.

## Results

Data from the 26 Brazilian federative units and the Federal District of Brazil (UFs) occurred in 2017 were studied in 2021. The UFs with the highest Maternal Mortality Ratio (MMR) in 2017 were in Pará (90.9 deaths per 100,000 live births), Maranhão and Tocantins (with 85.0 and 84.2 per 100,000 live births, respectively.) The highest rates of income inequality – measured by the Gini index – were observed in states of Amazonas and Bahia (Gini index of 0.60), while the lowest Human Development Index was observed in Alagoas (0.683) and the per capita income in Maranhão (R$387.70). In Acre, the highest Social Vulnerability Index (SCR of 0.374) was observed (Table 1).

**Table 1:** Maternal mortality ratio and income inequality, socioeconomic development and the vulnerability index in Brazil in 2017

Federative Unit	MMR	Gini Index	HDI	Income Per capita	SVI
Rondônia	69.1	0.46	0.725	619.23	0.191
Acre	48.9	0.57	0.719	498.02	0.374
Amazonas	64.0	0.60	0.733	558.03	0.327
Roraima	51.1	0.55	0.752	650.51	0.232
Pará	90.9	0.53	0.698	468.48	0.278
Amapá	45.5	0.59	0.74	605.04	0.253
Tocantins	84.2	0.50	0.743	610.38	0.24
Maranhão	85.0	0.54	0.687	387.70	0.349
Piauí	72.1	0.54	0.697	487.40	0.279
Ceará	65.7	0.56	0.735	538.22	0.272
Rio grande do Norte	75.7	0.53	0.731	550.17	0.283
Paraíba	62.6	0.56	0.722	601.71	0.292
Pernambuco	61.8	0.56	0.727	558.98	0.336
Alagoas	31.8	0.53	0.683	426.33	0.338
Sergipe	50.2	0.56	0.702	541.98	0.298
Bahia	64.7	0.60	0.714	566.60	0.298
Minas Gerais	44.8	0.50	0.787	804.61	0.207
Espirito santo	59.1	0.51	0.772	800.14	0.227
Rio de janeiro	73.9	0.52	0.796	960.11	0.284
São Paulo	56.6	0.53	0.826	1.134.12	0.241
Paraná	31.1	0.49	0.792	968.39	0.186
Santa Catarina	38.6	0.42	0.808	1.044.59	0.134
Rio Grande do Sul	37.4	0.49	0.787	1.073.13	0.209
Mato grosso do Sul	42.5	0.48	0.766	841.32	0.194
Mato grosso	59.4	0.47	0.774	809.58	0.227
Goiás	52.3	0.49	0.769	835.77	0.247
Distrito Federal	47.1	0.59	0.85	1.688.48	0.258

*MMR* Maternal Mortality Ratio, *HDI* Human Development Index, *SVI* Social Vulnerability Index.

**Fig. 1:**
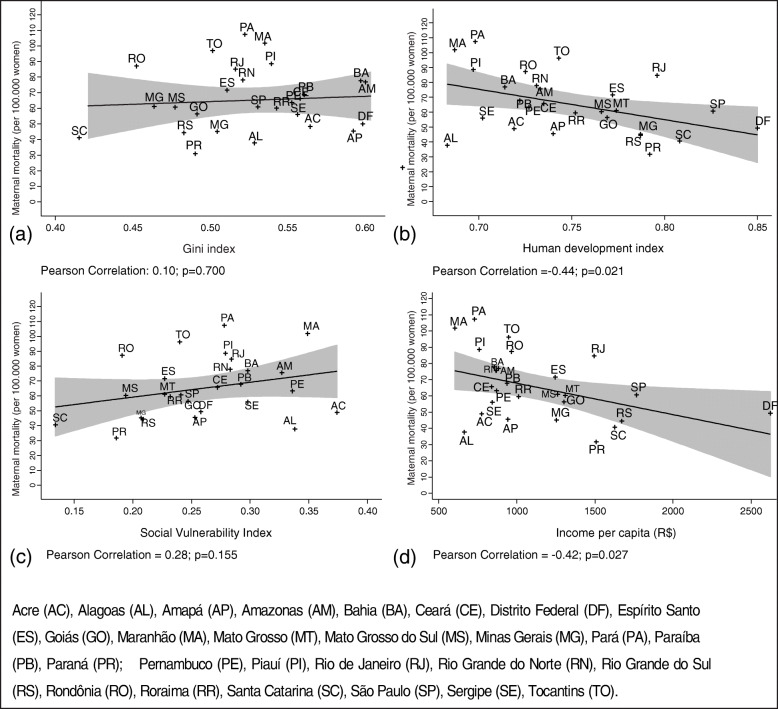
The relationship between socioeconomic determinants and maternal mortality ratio in Brazilian Federative units in 2017

When analyzing which socioeconomic determinants among those studied that are related to maternal mortality ratio rates, a higher HDI positive effect was observed for lower MMR (β = − 150.8; CI 95% -289.9 to − 11.7; *r*^2^ = 0.17; *p* = 0.035) (Fig. 1b), as well as a trend of higher MMR in relation to the SVI (β = 97.7; CI 95% -12.2 to 207.6; *r*^2^ = 0.12; *p* = 0.079) (Fig. 1d).

All exposure variables were included on the model I, to assess the role of each variable for the RMM when assessed together. In this model (model I), the adjusted R-square found was 0.05 and no variables presented statistical significance. However, when we used the stepwise forward method for variable selection (model II), where only variables with significance were included in model, only the per capita income was significant, where the MMR seems to reduce with the increase of this index (β = − 0.02; CI 95% -0.05 to − 0.002; *r*^2^ = 0.15; *p* = 0.028) (Table 2).

**Table 2:** Models to assess the relationship between maternal mortality ratio and Human Development Index, per capita income, Gini index and Social Vulnerability Index in Brazilian federative units in 2017

Variables	Maternal Mortality Ratio		
	β (95% CI)	adjusted *R*^2^	p*
Model I: All variables included			
Human Development Index	33.3 (− 429.1; 494.7)	0.05	0.882
Social Vulnerability Index	67.1 (− 139.5; 273.9)		0.507
Per capita income	−0.02 (− 0.09; 0.04)		0.487
Gini index	−22.9 (− 286.9; 374.8)		0.828
Model II: Stepwise selection			
Per capita income	−0.02 (− 0.05; − 0.002)	0.15	0.028

In view of perform a sensibility analysis, we perform both methods for variable selection (forward, backward and both methods together), but the results were the same.

## Discussion

The main results show a scenario where the MMR in Brazil in 2017 is mainly influenced by the per capita income. MMR analysis in the 27 states of the federation showed an inverse correlation between HDI and MMR and between per capita income and MMR. States with lower HDI values and lower per capita income, such as Maranhão, Piauí, and Pará, are among those with worse maternal mortality indicators. These findings are in line with other studies that point to higher maternal mortality in areas of poorer socioeconomic status [23–25].

Despite of income was an index related with HDI, in this study when assess the models with selection of variables significant, only the per capita income index was related to the MMR in Brazil, 2017. The differences of the per capita income used in this study with the income that compose the HDI was the per capita income assess average of the money for people in a place, while the income of HDI is related to the gross income per capita [10].

Several studies showed an inverse correlation between HDI and infant mortality [26]^.^ and HDI and circulatory diseases [27]. Other studies evaluating HDI around the world as a predictor of maternal and infant mortality concluded that it is strongly related to increased rates of these outcomes [28, 29]. The HDI is a composite indicator that aggregates three dimensions: health, education, and income. For the health dimension, the variable is life expectancy at birth. For education, it is the combination of two variables – mean years of study of the population aged 25 and over and expected years of study. For income, the variable is gross national income per capita [10].

Considering that most northeastern states still suffer from unacceptably high MMR, despite efforts to reduce them, the development of public policies aimed at the three domains included in HDI should be a priority.

It is worth noting that in all correlation analyses between maternal mortality and other socioeconomic variables, regional differences remained. These differences are reflexes of greater education and access to public services, showing the need for policies to decrease these inequities in the country, especially where there is greater inequality [12]. Regional differences in maternal mortality show that most of these deaths could be prevented.

The need to expand actions aimed at the most vulnerable populations is clear in the northern and northeastern regions because maternal mortality is an indicator that reflects social reality, and better health, education and income conditions influence this reality. A review about maternal mortality, which included 10-year articles from the PubMed electronic database, pointed out that conditions of vulnerability to maternal death are linked to teenage and old age pregnancy, cesarean delivery, interpartal interval less than 2 years, malnutrition, obesity and the racial issue, considering that the coefficient of maternal mortality is much higher in black women. Other highlighted vulnerability conditions in that review were low education, single women, unemployment, difficulty in accessing health services and the quality of care provided in these services [30]. The great majority of these vulnerabilities are related to the living conditions indicating social helplessness to the least favored women, resulting in maternal death resulting from multiple factors.

Additionally, the possible reasons for the absence of association between MMR and SVI may be related to the large number of indicators grouped in this index, to the focus of the analysis on a single year, and to the strong MMR variations from 1 year to another. SVI is a synthetic index that gathers sixteen indicators structured in three dimensions representing a set of assets, whose possession or deprivation determines the welfare conditions of populations in contemporary societies [15].

In this sense, studies evaluating the correlation of MMR with the three dimensions of SVI separately for a longer period may present different results from the ones in this study, considering that each one of them measures different areas of social vulnerability, and that they are not adequately reflected in the aggregate index.

Other point that needs to be discussed is the non-association between the income inequality and the MMR. The reason of inclusion of this index in this study was the absence of Brazilian data about this socioeconomic determinant and MRR. Thus, Vilda et al. [19] examined the association of income inequality and pregnancy-related mortality between black and white populations in the United States, reporting that this indicator was significantly associated with mortality only in black women.

Studies on the relation between other outcomes and income inequality in the female population were also conducted. Figueiredo and Adami [31] analyzed the correlation between this indicator and breast cancer mortality in Brazilian women using four indicators: Gini index, Palma index, Theil-L index, and the quintile ratio, concluding that increased breast cancer mortality is correlated with greater income inequality. They also reported worse indicators of breast cancer mortality rates and worse socioeconomic indicators in regions where there was high income inequality [12].

However, these research results are not unanimous. Other studies [32–34] corroborate this research, since they reported no evidence that income inequality, measured by the Gini coefficient, is associated with other health outcomes. Kondo et al. [35] conducted a multivariate meta-regression analysis of studies analyzing income inequality and mortality and self-assessment of health. The results showed that studies on income inequality in larger territorial areas, such as at the country level, presented a stronger association between this characteristic and health problems, in comparison with studies on small areas and populations, concluding that the heterogeneity between studies can be explained by variations in the size of the area or population in which income inequality was evaluated.

The findings of the multivariate analysis by Kondo et al. [35] may explain the results of the present study that analyzed the correlation between the Gini index and MMR in the 27 units of the federation and not in the entire country. Studies by Wilkinson and Pickett [36] and Barrozo [37] also corroborate the understanding that analyses performed in large areas are more favorable to the hypothesis that greater income inequalities are associated with lower health standards.

The particular strengths of the present study include analysis at the national level in search of correlations of socioeconomic indicators and MMR. This study helped to show that non-biological factors that are potentially modifiable may be associated with maternal mortality. The analysis used in this study offers additional information which can enrich the understanding of how economic and social inequalities related to the different maternal mortality rates within the country.

However, we understand that the results of this study should be interpreted with caution, and that some limitations must be considered. First, we can point out the fact that the period evaluated was only 1 year and that the mortality rates suffer great variations from 1 year to another. Secondly, synthetic indicators such as SVI and HDI, measure performance in different socioeconomic areas that are not adequately reflected in the aggregate index. In addition, we understand that the use of other variables related the MMR can be changed the direction and strength of these findings, and that their exclusion can lead to false interpretations, but we emphasize that the exclusion strategy reflects the focus of this study on the socioeconomic determinants of maternal mortality.

It is understood that the entire research is not limited to the results included and, in view of the limitations of the present study, it is recommended that future work be carried out in support of the findings of this research and that include other important variables that influence the maternal mortality, such as health behavior and biological factors.

Thirdly, this study was a cross-sectional analysis at the level of states in the federation and therefore it is not possible to draw causal inferences from the results. As we use States as the unit of analysis, we cannot provide any information about the variation within each Brazilian municipality, and for this cause our sample size is small and the finding from this study cannot be generalized. Thus, the results of this study should be considered as a first description of the relationship between socioeconomic inequalities and maternal mortality. In future studies, the use of disaggregated indicators should be considered in the analysis of this association.

This study, analyzing socioeconomic aspects, pointed out the inequalities between Brazilian states, identifying those who are in vulnerable conditions and how these vulnerabilities are related to maternal mortality. The political impact of this study lies in the fact that it can contribute to subsidize the organization and the planning of actions and multisectoral strategies to face the phenomenon of maternal death.

Therefore, with this analysis we hope to contribute to provide decision makers with the type of information necessary for the establishment of priority public policies with the potential to change the vulnerability of the female population, in other words, policies that transcend the health sector and seek mitigate socioeconomic inequalities between Brazilian states that reflect maternal mortality rates.

## Conclusion

The results of the present article show that the MMR is higher in the northern and northeastern states of the country, and that the per capita income have a negative association MMR in the different states of Brazil, but seems canceled because of the other socioeconomic determinants related to the poor live conditions.

## Data Availability

The databases used in this research are public, not requiring prior authorization for access and use.

## Abbreviations

MMR: Maternal Mortality Ratio
HDI: Human Development Index
SVI: Index and Social Vulnerability Index
PROADESS: Health System Performance Evaluation Project
IPEA: Institute of Applied Economic Research
UFs: Federative Units
